# Ultrafast Optical Analysis and Control of Spectral Flatness in Cavity-Less Electro-Optic Combs

**DOI:** 10.3390/mi17030350

**Published:** 2026-03-12

**Authors:** Xin Chen, Hongyu Zhang, Meicheng Fu, Huan Chen, Yi Zhang, Yao Xu, Mengjun Zhu, Wenjun Yi, Qi Yu, Junli Qi, Qi Huang, Yubo Luo, Xiujian Li

**Affiliations:** 1 College of Science, National University of Defense Technology, Changsha 410073, China; chenxin_cx@nudt.edu.cn (X.C.); zhanghya1006@163.com (H.Z.); chenhuan11@nudt.edu.cn (H.C.); yizhangan1er@163.com (Y.Z.); xuyao0611621@163.com (Y.X.); yiwenjun@nudt.edu.cn (W.Y.); yuqi10@nudt.edu.cn (Q.Y.); qijunli_r@163.com (J.Q.); huangqi@nudt.edu.cn (Q.H.); luoyubo24@nudt.edu.cn (Y.L.); 2 School of Information and Electronics Engineering, Hunan City University, Yiyang 413000, China; mjzhu16@163.com

**Keywords:** microwave photonics, cavity-less electro-optic combs, spectral flatness, extinction ratio, spectral interference

## Abstract

The cavity-less electro-optic combs (EOCs), recognized for exceptional tunability, stability and high power, are a crucial enabler for the fields such as optical communications, precision measurement and metrology, and microwave photonics. This work systematically investigates the fundamental physical factors that govern the spectral flatness via ultrafast measurements and modeling simulations. The ultrafast analysis results demonstrate that, the finite effective modulation extinction ratio of the electro-optic intensity modulators will result in generation of coherent spectral components with identical frequencies but varying phases and amplitudes in ultrashort temporal scale, finally lead to remarkable spectral interference and further intensity fluctuations across the combs spectrum. Furthermore, the established mathematical relationship between the spectral flatness and the modulation extinction ratio of the intensity modulators exhibits a nonlinear dependence up to the third order. Cascading intensity modulators has been exploited to mitigate the spectral interference and improve the modulation extinction ratio, which has been verified by using home-made high sensitive autocorrelator and frequency-resolved optical gating (FROG), and finely spectral flatness of 0.54 dB among 11 lines has been achieved, which recognized for the first time that modulation extinction ratio related spectral interference phenomenon play a subtle role in EOCs generation. Furthermore, photonic analog-to-digital converters (PADCs) have been investigated and an obvious enhancement in signal-to-noise-and-distortion (SINAD) is achieved, These findings will provide crucial theoretical and experimental support for optimizing EOCs performance, and advance the development and application.

## 1. Introduction

As a major type of optical frequency combs (OFCs) [1,2,3,4], with highly flexible tunability, stability and high per comb line power up to milliwatt level, the cavity-less electro-optic combs (EOCs) with flat broadband spectrum have attracted many applications [5,6], including single-comb spectroscopy, dual-comb spectroscopy, optical communications and photonic analog-to-digital converters (PADCs) [1,7,8,9,10,11]. Basically, besides the driving RF signal performance, the performance of cavity-less EOCs is directly determined by the intrinsic properties of the electro-optic modulators, including the bandwidth and the modulation extinction ratio. For decades, numerous methods have been proposed to improve spectral flatness of the cavity-less EOCs based on fiber [12,13,14], and integrated modulators [15,16]. For example, R. Wu et al found that the flat spectrum can be achieved by adding an extra suitable RF second harmonic into the phase modulator (PM) [17], while Y. Dou et al. found that the spectral flatness can be improved by setting the DC bias below the linear modulation point [18]. Meanwhile, Z. Xie et al. used a dual-parallel Mach–Zehnder modulator (DPMZM) to improve the spectral flatness [19]. Y. Guo et al. used a polarization modulator (PolM) and a first-order Butterworth band-stop filter to generate ultra-flat optical frequency combs (OFCs) [20].

The theoretical model of the cavity-less EOCs has been analyzed with time-lens technologies [21,22], in which a sharp-pulse with high temporal contrast generated by the intensity modulator (IM) is mapped into a flat-top-spectrum by the PM with a large near-parabolic phase modulation [22,23]. Although numerous methods have been demonstrated to achieve flat spectrum based on time-lens model, the primary consideration is the phase modulation performance, i.e., tailoring the driving RF of PMs. Cascading IMs has also been proposed to improve the spectral flatness, however, its physical essence remains the pursuit of a more parabolic phase modulation [17]. In this work, a modulation extinction ratio related spectral interference phenomenon has been investigated for the first time, and the results show that the performance of IMs also plays a vital role in achieving flat EOCs.

Herein, by cascading IMs to vary the modulation extinction ratio, we analyze the generated pulses in both the time and frequency domains by the ultrafast measurements and modeling. The results demonstrate that the spectral flatness of cavity-less electro-optic combs (EOCs) is primarily governed by spectral interference. This interference originates from the coherent superposition of background DC stray light leaked due to the finite modulation extinction ratio of the IMs. Guided by this insight, we improved the spectral flatness of the central 11 combs from 1.16 dB to 0.54 dB solely by increasing the modulation extinction ratio. The excellent agreement between experiments and simulations validates our model, which establishes a clear, up-to-third-order nonlinear relationship between spectral flatness and the modulation extinction ratio. Our findings can also be extended to other wavelength regions based on electro-optic modulators [24].

## 2. Ultrafast Measurements of EOCs

**Figure 1:**
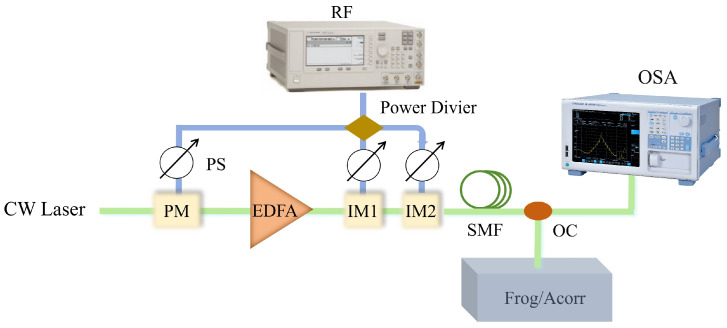
Setup for cavity-less EOCs measurements and simulations. RF, radio frequency source; PS, RF phase shifter; PM, phase modulator; EDFA, erbium-doped fiber amplifier; IM, intensity modulator; SMF, single-mode fiber; OC, optical coupler; OSA, optical spectrum analyzer; FROG/Acorr, frequency-resolved optical gating setup/autocorrelator.

The setup for analyzing the cavity-less EOCs is illustrated in Figure 1. A CW laser with wavelength of 1547.7 nm and power of 16 dBm (∼39.8 mW) works as the laser source. The light is firstly modulated by three PMs, here we set the total modulation index to 13.44 rad ($m=\pi \cdot V_{RF}/V_{\pi }$, where $V_{RF}$ and $V_{\pi }$ is the applied voltage and thehalf-wave voltage of the PM respectively). The number of IMs in the setup will be different for various extinction ratio conditions. The driving RF frequency of the modulators is 5.2 GHz. All the IMs are controlled by the high-precision automatic bias controller. The single-mode fiber (SMF) with a length of 3.8 km has been chosen for compensating the frequency chirp based on the simulations and experiments [25]. To measure the ultrashort pulse finely, the temporal ultrafast characteristics of the output pulse are characterized by an home-made autocorrelator with a high sensitive single-photon detector (PicoHarp 300, PicoQuant, Berlin, Germany) [26], and a high-precision frequency-resolved optical gating (FROG) setup with a sensitive grating spectrometer (Horiba FHR-1000, HORIBA, France with a liquid nitrogen-cooled Symphony-II detector) [25,27]. The EOCs’ spectrum is measured by an optical spectrum analyzer (OSA, AQ6380).

**Figure 2:**
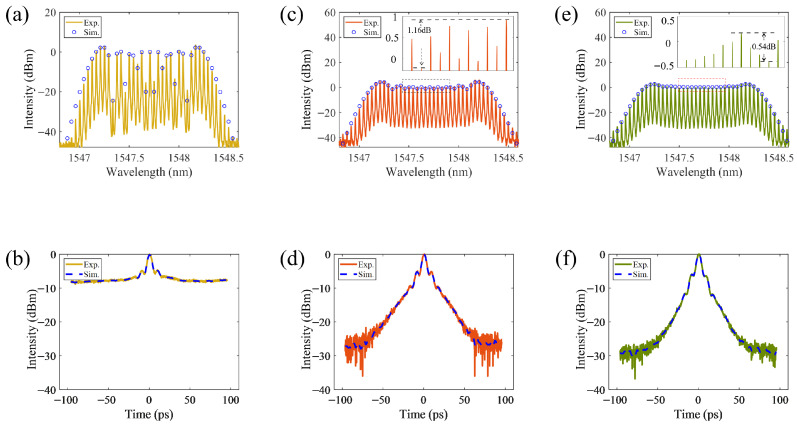
The output spectral and ultrafast temporal profiles for case #1, #2 and #3 respectively. (**a**,**c**,**e**) the experimental (Exp.) and simulated (Sim.) spectral profiles, (**b**,**d**,**f**) the experimental (Exp.) and simulated (Sim.) ultrafast temporal profiles measured with autocorrelation method.

We set three cases for the measurements and the simulations, i.e., case #1 (only with three PMs), case #2 (with three PMs and one IM) and case #3 (with three PMs and two IMs). The experimental (Exp.) and the simulational (Sim.) spectral profiles of the output pulses are shown in Figure 2a, Figure 2c, and Figure 2e, for case #1, case #2 and case #3 respectively. Obviously, the difference among the three cases is remarkable. The spectral combs are rough for case #1, relatively flat and with two high “rabbit ears” for case #2, while very smooth flat and without obvious “rabbit ears” for case #3. For the case #2 and #3, the flatness (=$I_{max}-I_{min}$) of the central 11 combs around wavelength of 1547.7 nm is 1.16 dB and 0.54 dB respectively, shown as the zoom-in inserts in Figure 2c and Figure 2e with standard deviations of the strength of the whole central 23 combs about 1.68 and 0.577 respectively.

Meanwhile, we perform ultrafast measurements and corresponding simulations to analyze the ultrafast temporal pulse profiles using a high-dynamic-range sensitive autocorrelator as shown in Figure 1, in which the modulation coefficient of the PMs was 13.44, and the group velocity dispersion (GVD) and the third-order dispersion (TOD) of the SMF was −0.0217 ps^2^/km and 0.015 ps^3^/km respectively, and the modulation extinction ratio is 23 dB and 30 dB for case #2 and #3 respectively. The output optical power from the SMF was controlled to approximately 6 dBm by an EDFA. The yellow line in Figure 2b, the red line in Figure 2d, and the green line in Figure 2f represent the autocorrelation measurements for case #1, #2 and #3, with contrast ratio of 8 dB, 27.36 dB, and 30.81 dB, respectively.

**Figure 3:**
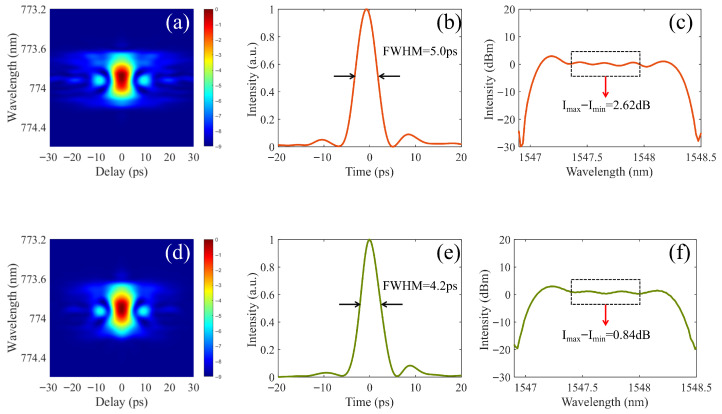
FROG measurements of EOCs for case #2 and #3. (**a**,**d**) the measured FROG traces, (**b**,**e**) the reconstructed ultrafast temporal pulse profiles, (**c**,**f**) the corresponding reconstructed pulse spectra. (**a**–**c**) for case #2, (**d**–**f**) for case #3.

Further analysis of Figure 2 demonstrates that, higher modulation extinction ratio of the IMs will be helpful for achieving better spectral flatness and sharper pulses, i.e., higher contrast ratio and smaller FWHM. Besides the autocorrelation measurements emphasizing on measuring the ultrashort pulse contrast ratio, the FROG measurements are performed for case #2 and case #3, which emphasize on achieving the ultrafast temporal pulse profile in sub-femtosecond temporal resolution and sub-nanometer spectral resolution simultaneously. The measured FROG traces are shown in Figure 3a,d, the reconstructed temporal pulse profiles are shown in Figure 3b,e, and the corresponding reconstructed spectral pulse profiles are illustrated in Figure 3c,f.

In comparison to Figure 3b (FWHM = 5.0 ps), the pulse in Figure 3e is shorter (FWHM = 4.2 ps), and the spectral flatness in Figure 3f is significantly better than that in Figure 3c, which obviously indicates that pulse performance for case #3 is better than that for case #2. The pulse performance improvement from case #2 to case #3 is confirmed to be due to the reduction of stray light by higher modulation extinction ratio of the cascaded IMs. Here the pulse performance improvements also demonstrate the fundamental physical factor that determines the output spectral flatness of the cavity-less EOCs, i.e., the ultrafast coherent superposition of the stray background light components and the pulse peak light components inside the pulse may lead to remarkable spectral fluctuations over the broad band. Therefore, under consistent conditions for other components, by controlling the modulation extinction ratio of the IMs, we achieve high pulse contrast ratio with fine spectral flatness.

## 3. The Relationship of Spectral Flatness vs. Extinction Ratio of IM

**Figure 4:**
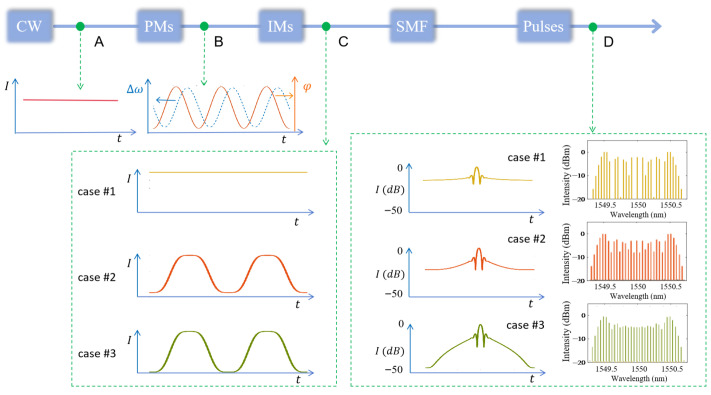
Opticaltransmission chain diagram of EOCs for the three cases.

Based on the setup shown in Figure 1, we further performed the ultrafast simulations for more check points as shown in Figure 4, with the evolution of the pulse’s profiles at various points along the light flowing.

When the CW laser light (as the temporal intensity profile of point A in Figure 4) is modulated by the three cascaded PMs driven with the suitable phase-shifted RF signal, the sinusoidal phase and the corresponding frequency chirp (Δω) will be loaded onto it (shown as the lines out of point B in Figure 4). Then, depending on the applied RF signal and the corresponding phase shift, and the DC bias for the IMs, the light passes through the IMs will present different intensity curves (shown as the lines out of point C in Figure 4 for the three cases). The output intensity profiles vary significantly with the modulation extinction ratio of the IMs, with the contrast ratio decreasing in the order of case #3 (highest) > case #2 (elevated valley) > case #1 (flat baseline). Finally, the distinct pulse profiles are observed at the SMF output for the three cases, which is confirmed by the autocorrelation simulations of the final EOCs output at point D (Figure 4).

Obviously, according to the output at point D in Figure 4, due to the time-space duality [28], the dispersion compensation of the SMF will generate pulses even though no pulse-shape intensity modulation other than sinusoidal phase modulation applied on the laser light for case #1. However, the pulses will be with poor quality, including low contrast ratio, large background DC stray light, and poor spectral flatness over the whole band. For case #2, when the IM (with an modulation extinction ratio of 23 dB) is driven solely by a high-power sinusoidal RF signal, the resulting pulse quality of the output frequency comb is much improved, with superior spectral flatness in the central region compared to case #1. Furthermore, in case #3, two cascaded IMs with a total modulation extinction ratio of 30 dB are driven by the same sinusoidal RF signal as case #2, which results in significantly improved pulse performance, particularly a much better spectral flatness in the central wavelength region than that of both cases #1 and #2. All the simulated results confirm that, the modulation extinction ratio of the IMs significantly determines the output comb quality in terms of pulse contrast ratio and spectral flatness.

**Figure 5:**
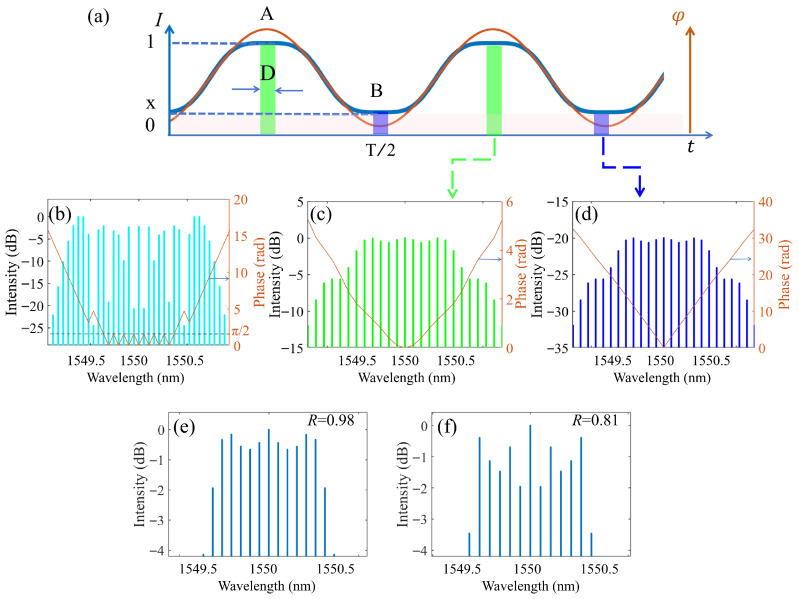
Simulation model and results for EOCs output optimizing. (**a**) Diagram of modulation parameters for spectral interference simulations, (**b**) the combs with phase profile generated by the PMs, $E_{PM}\left(f\right)$, (**c**) the combs of the location corresponding to the green line $E_{A}\left(f\right)$, (**d**) the combs of the location corresponding to the blue line $E_{B}\left(f\right)$, (**e**) R=0.98, the final combs of E(f), and (**f**) R=0.81, the final combs of E(f). Here we consider the combs are the Fourier transform of the temporal intensity distributions.

Analysis of Figure 2 through Figure 4 reveals that the modulation extinction ratio of the IMs is a critical determinant of the EOCs’ temporal and spectral quality. The ultrafast coherent simulation model, illustrated in Figure 5, is based on the coherent superposition of the pulse and the stray background light. It specifically accounts for the modulation extinction ratio of the IMs, defined as the ratio of the peak intensity to the background DC stray light intensity in the valley. As shown in Figure 5a, when the modulaiton extinction ratio of the modulator is not ideal, the intensity profiles of the modulated pulse are represented by the blue line, respectively. The flat-top pulse portion with a width of D was extracted for investigation, as depicted by the green rectangle. The temporal intensity profile of this pulse can be expressed as A(t). The finite extinction ratio results in a background of DC stray light in the pulse valley (represented by the blue rectangle), the intensity of which can be expressed as B(t). When the width of the rectangular window is D, the spectral flatness of the pulse generated by a single IM within the rectangular window is the highest. In the simulations, the amplitude of A(t) and B(t) is set to 1 and (1−R)/(1+R), respectively, in which *R* represents the modulation extinction ratio of the IMs.

A(t) and B(t) are expressed in Equation (1), where *T* is the period of the RF signal.

(1) $$\left\{\begin{array}{c} A\left(t\right)=rect\left(\frac{t}{D}\right)*\sum_{n=-\infty }^{n=+\infty }\delta \left(t-nT\right),\\ B\left(t\right)=\sqrt{\frac{1-R}{1+R}}\cdot rect\left(\frac{t-\frac{T}{2}}{D}\right)*\sum_{n=-\infty }^{n=+\infty }\delta \left(t-nT\right) \end{array}\right.$$

When the monochromatic light $E_{in}\left(t\right)=e^{-i\omega_{0}t}$ is modulated by the PMs with RF frequency of $\omega_{RF}$, the complex amplitude will be $E_{PM}\left(t\right)=E_{in}\left(t\right)e^{-imcos(\omega_{RF}t)}$. As evident from Figure 5b (orange line), the phase difference between each tooth of the light modulated exclusively by the PMs is π/2. The light is then subsequently modulated by the IMs. The output components, corresponding to the green (A) and blue (B) rectangles in Figure 5a, have complex amplitude given by:

(2) $$\left\{\begin{array}{c} E_{A}\left(t\right)=E_{PM}\left(t\right)\cdot rect\left(\frac{t}{D}\right)*\sum_{n=-\infty }^{n=+\infty }\delta \left(t-nT\right),\\ E_{B}\left(t\right)=E_{PM}\left(t\right)\cdot \sqrt{\frac{1-R}{1+R}}\cdot rect\left(\frac{t-\frac{T}{2}}{D}\right)*\sum_{n=-\infty }^{n=+\infty }\delta \left(t-nT\right) \end{array}\right.$$

And,

(3) $$\left\{\begin{array}{c} E_{A}\left(f\right)=E_{PM}\left(f\right)*\left[\sum_{n=-\infty }^{\infty }f_{RF}\;\delta \left(f-nf_{RF}\right)\cdot D\cdot \mathrm{sinc}\left(fD\right)\right],\\ \begin{array}{cc} E_{B}\left(f\right)= & \;E_{PM}\left(f\right)*\left[\sum_{n=-\infty }^{\infty }f_{RF}\;\delta \left(f-nf_{RF}\right)\cdot \sqrt{\frac{1-R}{1+R}}\cdot D\cdot \mathrm{sinc}\left(fD\right)\;e^{-j\frac{pifT}{D}}\right] \end{array} \end{array}\right.$$

where, $E_{A}\left(f\right)$ and $E_{B}\left(f\right)$ is the short-time Fourier transform of $E_{A}\left(t\right)$ and $E_{B}\left(t\right)$, respectively, as shown in Figure 5c and Figure 5d; $f_{RF}$ determines both the frequency of the RF signal and the resulting comb spacing. The intensity of the green rectangle is much larger than that of the blue rectangle in Figure 5a. Similarly, this contrast is maintained in their corresponding Fourier transform shown in Figure 5c,d, i.e., $E_{A}\left(t\right)\gg E_{B}\left(t\right)$ and $E_{A}\left(f\right)\gg E_{B}\left(f\right)$. The total light intensity in the spectral domain E(f) is modeled as the coherent superposition of $E_{A}\left(f\right)$ and $E_{B}\left(f\right)$, as described by Equation (4).

(4) $$E\left(f\right)=|E_{A}\left(f\right)+E_{B}{\left(f\right)|}^{2}$$

Unlike a direct intensity addition, coherent superposition involving constructive and destructive interference produces a final frequency comb that differs markedly from the peak-wave comb of the green rectangle in Figure 5a. Furthermore, A lower extinction ratio (R=0.81 compared to R=0.98) results in degraded spectral flatness of the final frequency comb, as shown in Figure 5e,f.

The spectral flatness of the frequency comb is ultimately governed by the extinction ratio of the IMs. This is because the components at the spectrum center share the same frequency but have different phases (Figure 5c–e), which make their coherent superposition cause interference fluctuations [29]. Obviously, a higher extinction ratio will lead to near zero intensity of the background of DC stray light and finally mitigate this interference fluctuations.

**Figure 6:**
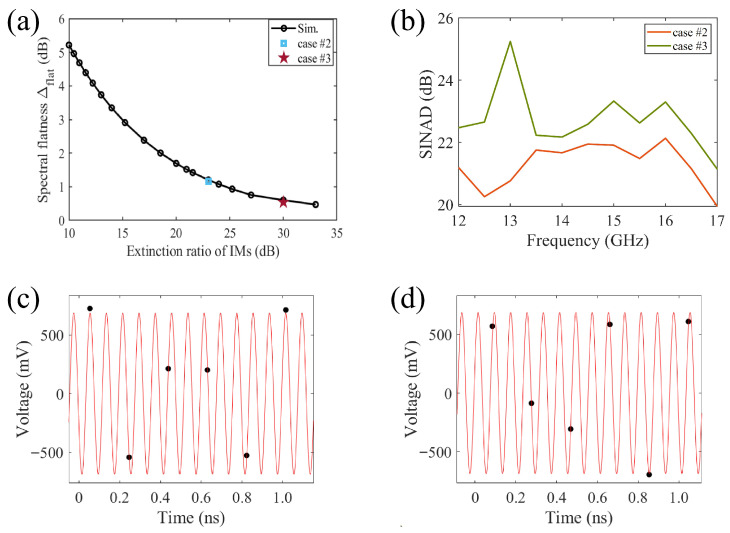
(**a**) Spectral flatness of the EOCs vs extinction ratio of IMs, (**b**) the measured PADCs SINAD for case #2 and #3, (**c**) analog-to-digital conversion results of a 12.5 GHz single-tone signal for case #2, (**d**) analog-to-digital conversion results of a 12.5 GHz single-tone signal for case #3.

We further investigate the mathematical relationship between the extinction ratio of the IMs and the spectral flatness of the combs. Considering the electro-optic modulation contrast of the IMs to be $-10log_{10}\left(\left(1-R\right)/\left(1+R\right)\right)$, we can achieve the simulation results shown in Figure 6a by ranging *R*, where the measurements for case #2 and #3 are included. The flatness of the measured combs for case #2 and #3 is 1.17 dB and 0.54 dB, and the flatness of the simulated results corresponding to the same contrast (R=0.99 and 0.998) is 1.204 dB and 0.603 dB respectively. By nonlinear fitting the data from Figure 6a, we obtain an expression for the functional dependence of the comb spectral flatness ($\Delta_{flat}$, in dB) on the IM extinction ratio, up to the third order, as shown in Equation (5), where *a* = 0.00034, *b* = 0.33, *c* = 1.1, *d* = 13.

(5) $$\begin{array}{c} \Delta_{flat}=aR^{3}+bR^{2}+cR+d \end{array}$$

## 4. Improvement of PADCs

Recently, the frequency response enhancement of the PADCs through improving the flatness of the pulses’ optical spectrum [30] and optical power spectrum [31] has been demonstrated. To further evaluate the impact of the improved flatness of the optical frequency comb on our subsequent research, we conducts a series of PADCs experiments utilizing the EOCs under conditions of case #2 and case #3 for RF signal sampling. The Ku-band RF signal for measurements is widely used in satellite broadcasting, radar systems, and military communications. The process begins with an erbium-doped fiber amplifier (EDFA) boosting the pulse train from the cavity-less optical source. These amplified pulses are subsequently applied to a broadband MZM (KG-AM-40G) for sampling the single-tone microwave signal (Agilent E8267D). Following detection by a photodetector (PT10XGC), the sampled optical signal is digitized by an ADC (RFSoC 47DR), which features an analog bandwidth of 6 GHz.

The measured results for case #2 and case #3 are shown in Figure 6b–d, in which the RF signal is set to the same power level for all the measurements. We use the three-parameter fitting method to calculate the SINAD of the collected data. where a sine wave with the form Acos(2πft)+Bsin(2πft)+C was fitted to the acquired data by minimizing the squared error [32]. The signal power was obtained from the fitted amplitudes as $(A^{2}+B^{2})/2$, and the noise-plus-distortion power was calculated from the variance of the residual. Figure 6b presents the measured SINAD at different input signal frequencies by using the two optical pulse sources. Meanwhile, we selected the sampling results at 12.5 GHz for display, as shown in the Figure 6c,d. The black circles represent the sampling points, while the red curve depicts the standard single-frequency signal calculated using the three-parameter fitting method. Obviously, the SINAD achieved with the EOCs under case #3 is higher than that of case #2, showing an improvement between 0.51 dB and 4.5 dB. This confirms that improving the spectral flatness of the EOCs directly enhances the SINAD of the PADCs system.

## 5. Conclusions

To optimize the spectral flatness of cavity-less electro-optic combs (EOCs), we constructed an experimental system incorporating cascaded phase modulators (PMs) and intensity modulators (IMs) with variable extinction ratio. Through ultrafast measurements and modeling simulations, we identified the effective modulation extinction ratio of the IMs as the critical factor governing the temporal and spectral quality of the EOCs. A finite extinction ratio results in background DC stray light with coherent spectral components at identical frequencies but differing phases and amplitudes within the combs center. This leads to significant spectral interference, inducing intensity fluctuations across the entire comb spectrum and ultimately degrading comb performance.

Thus, spectral interference arising from the finite extinction ratio of the IMs is established as the fundamental physical mechanism determining the cavity-less EOCs output spectral flatness. Our findings confirm that enhancing combs flatness requires minimizing stray light through higher extinction ratios achieved via cascaded IMs. Based on experimental and simulated data, we established a clear mathematical relationship between spectral flatness and modulation extinction ratio, which follows a third-order nonlinear dependence.

Guided by this physical insight, we improved the spectral flatness of the central 11 combs of a cavity-less EOCs from 1.16 dB to 0.54 dB solely by increasing the extinction ratio. These results demonstrate that enhancing the effective modulation extinction ratio through cascaded IMs is an effective method for improving spectral flatness. Experimental validation shows that this optimization directly translates to an increased SINAD in PADCs operating in the Ku-band. Our work provides theoretical and experimental foundations for optimizing cavity-less EOC performance, advancing the development and application of high-performance PADC technology in broadband RF signal measurement.

## Data Availability

The original contributions presented in this study are included in the article. Further inquiries can be directed to the corresponding authors.
